# Invasive Aspergillosis Masquerading as Multiple Pulmonary Nodules: A Challenging Diagnosis

**DOI:** 10.7759/cureus.8186

**Published:** 2020-05-18

**Authors:** Mamta Chhabria, Sharini Venugopal, Venkata Satish Pendela, Nagesh Jadhav

**Affiliations:** 1 Internal Medicine, Rochester General Hospital, Rochester, USA

**Keywords:** pulmonary aspergillosis, invasive aspergillosis, immunodeficiency, fungal lung infection

## Abstract

Invasive pulmonary aspergillosis (IPA) is an aggressive fungal infection of the lungs characterized by tissue invasion by fungal hyphal elements. The definitive diagnosis is challenging because it relies on histopathological demonstration of fungal elements, and these days clinicians are relying more on bronchoalveolar lavage (BAL) cultures and serum biomarkers (galactomannan and beta-D-glucan). We would like to emphasize through our case the necessity to keep a high index of suspicion for IPA despite negative cultures and serum biomarkers in immunosuppressed patients and consider surgical biopsy early.

## Introduction

Invasive pulmonary aspergillosis (IPA) is a rare fungal infection that thrives in the setting of immunosuppression. Contributing risk factors include immunosuppression associated with hematological malignancies, hematopoietic stem cell transplant, solid organ transplant, severe neutropenia, and use of high doses of glucocorticoids. Diagnosis can be challenging with bronchoalveolar lavage cultures and serum biomarkers (galactomannan and beta-D-glucan assays) being negative, necessitating biopsy for histopathological confirmation [1]. We present here a case of IPA that evaded diagnosis for nearly three months.

## Case presentation

**Figure 1:**
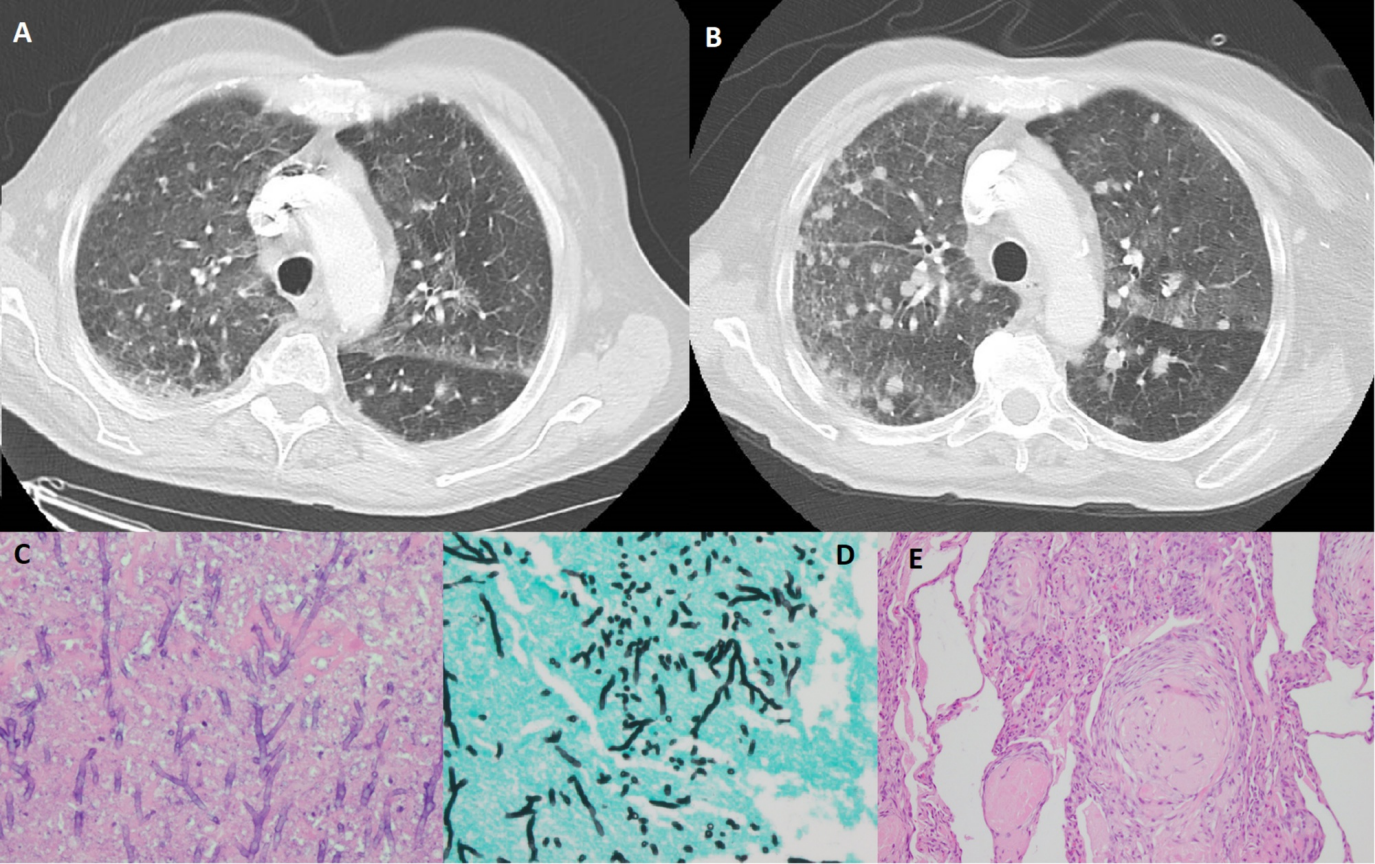
Imaging and pathology. (A) First CT scan demonstrating pulmonary nodules, septal thickening, and ground glass opacities. (B) Pulmonary nodules increased in size and number two months after first CT. (C) Numerous branching septate hyphae consistent with Aspergillus species. (D) Numerous septate hyphae showing airspace invasion (Gomori methenamine silver stain). (E) Granuloma formation.

An 81-year-old man with a history of chronic lymphocytic leukemia (CLL) and acquired hypogammaglobulinemia on monthly IV immunoglobin injections, a recent history of Nocardia brain abscess for which he received a long course of trimethoprim-sulfamethoxazole/amoxicillin-clavulanate presented with a four-month history of worsening shortness of breath. On examination, he was hypoxic on room air with saturation improving to 90% on supplemental oxygen. He was also noted to have bilateral rales to the midlung fields. His vital signs were normal. A CT chest revealed numerous pulmonary nodules, ground glass opacities, and interlobular septal thickening (Figure 1, Tile A). He underwent bronchoscopy with bronchoalveolar lavage (BAL) with transbronchial needle biopsy (TBNB). Sputum and BAL cultures grew Pseudomonas, and TBNB pathology showed focal organizing pneumonia. He received piperacillin-tazobactam, switched to levofloxacin upon discharge to complete seven days along with a prednisone taper. Fungal, viral, acid-fast bacteria (AFB) cultures, serum, and BAL galactomannan were negative at that time. The patient reported symptomatic improvement in dyspnea upon discharge but started developing shortness of breath again within a week of cessation of the steroid taper.

The patient returned two months later complaining of cough, clear watery sputum, rhinorrhea, and worsening dyspnea with increasing oxygen demands. His examination revealed diffuse crackles throughout both lungs, and hypoxia which improved with high flow oxygen. CT chest done revealed pulmonary nodules, which were increasing in size and number (Figure 1, Tile B). He underwent a repeat bronchoscopy (because he initially refused surgery), and fungal, viral, AFB cultures, serum and BAL galactomannan were negative again.

 There was a concern that his pulmonary nodules represented progression of CLL or a new primary malignancy. He agreed to undergo video-assisted thoracoscopic surgery with multiple wedge resections. The final pathology of these specimens revealed necrotizing granulomas and numerous septate fungal hyphae consistent with Aspergillus species (Figure 1, Tiles C,D, and E), and cultures grew Aspergillus fumigatus. The patient was started on antifungal voriconazole, later switched to aisavuconazole with a slow taper of steroids. He started to demonstrate a gradual improvement in oxygen demands and dyspnea. The patient experienced a protracted hospitalization because of the development of pseudomonas pneumonia and two intubations for which he needed additional antibacterial therapy. He was eventually weaned down to two liters supplemental oxygen therapy and discharged home.

## Discussion

Invasive pulmonary aspergillosis is an opportunistic fungal infection with a high mortality rate. The diagnosis is often delayed due to nonspecific symptoms and poor sensitivity of laboratory tests. Tissue biopsy is the diagnostic gold standard but it is often difficult to obtain a sample especially in unstable critically ill patients.

Bronchoscopy with BAL fungal cultures to diagnose IPA are highly specific tests; however, they have low sensitivity, with culture yields being as low as 30%-40% according to some studies [2-4]. In recent years, galactomannan testing is considered a powerful diagnostic test for IPA. It has a sensitivity of 86.4% and a specificity of 90.7% for an optical density index (ODI) of >= 0.8 while the specificity increases to 100% for an ODI >=3.0 [5]. However, even galactomannan testing was negative twice in our patient.

Our case reiterates that a high index of suspicion is necessary to successfully diagnose pulmonary aspergillosis. In this case, there were other differential diagnoses which could look similar radiologically including progression of his CLL, new primary malignancy, or metastatic disease. A surgical biopsy eventually clinched the diagnosis and put the patient on the right treatment path which leads us to conclude that surgical biopsy should be considered early in cases where invasive aspergillosis is in the top differentials. This would prevent delays in the administration of targeted antifungal agents. Further studies are required to determine the current sensitivities of the different tests available for diagnosis of invasive aspergillosis. 

## Conclusions

Our case highlights a common scenario in which diagnosis of invasive aspergillosis is delayed because a surgical biopsy was pursued late. We conclude that in patients who are immunocompromised, surgical biopsy should be considered early even if initial BAL cultures and serum biomarkers (galactomannan, beta-D-glucan) are negative. 
